# Outbreak of Intermediate Species *Leptospira venezuelensis* Spread by Rodents to Cows and Humans in *L. interrogans*–Endemic Region, Venezuela

**DOI:** 10.3201/eid3008.231562

**Published:** 2024-08

**Authors:** Lizeth Caraballo, Yaritza Rangel, Armando Reyna-Bello, Mariana Muñoz, Roque Figueroa-Espinosa, Carlos E. Sanz-Rodriguez, Elba Guerrero, Carmen Luisa Loureiro, Qingyun Liu, Howard E. Takiff

**Affiliations:** Instituto Venezolano de Investigaciones Científicas, Caracas, Venezuela (L. Caraballo, Y. Rangel, M. Muñoz, R. Figueroa-Espinosa, C.E. Sanz-Rodriguez, E. Guerrero, C.L. Loureiro, H.E. Takiff);; Universidad Nacional Experimental Simón Rodríguez, Caracas (A. Reyna-Bello);; Harvard T.H. Chan School of Public Health, Boston, Massachusetts, USA (Q. Liu)

**Keywords:** leptospirosis, *Leptospira*, *Leptospira venezuelensis*, *Leptospira interrogans*, liver disease, kidney disease, Weil’s disease, intermediate clade, intermediate species, cows, humans, bacteria, zoonoses, Venezuela

## Abstract

Leptospirosis is a common but underdiagnosed zoonosis. We conducted a 1-year prospective study in La Guaira State, Venezuela, analyzing 71 hospitalized patients who had possible leptospirosis and sampling local rodents and dairy cows. *Leptospira rrs* gene PCR test results were positive in blood or urine samples from 37/71 patients. *Leptospira* spp. were isolated from cultured blood or urine samples of 36/71 patients; 29 had *L. interrogans,* 3 *L. noguchii*, and 4 *L. venezuelensis*. Conjunctival suffusion was the most distinguishing clinical sign, many patients had liver involvement, and 8/30 patients with *L. interrogans* infections died. The *Leptospira* spp. found in humans were also isolated from local rodents; *L. interrogans* and *L. venezuelensis* were isolated from cows on a nearby, rodent-infested farm. Phylogenetic clustering of *L. venezuelensis* isolates suggested a recently expanded outbreak strain spread by rodents. Increased awareness of leptospirosis prevalence and rapid diagnostic tests are needed to improve patient outcomes.

Leptospirosis, one of the most common zoonoses worldwide, (*1*,*2*) is caused by *Leptospira* spp. In humans, its most severe, multiorgan, potentially fatal form is known as Weil’s disease (*3*). *Leptospira* can also infect animals, such as cattle, sheep, cats, and dogs. Rodents are the reservoir for most *Leptospira* spp.; rodent kidneys can become colonized with *Leptospira* and chronically shed the bacteria in urine. Except for occupational or recreational exposure, leptospirosis generally occurs in residents of marginal, rodent-infested areas, often in coastal regions of tropical countries (*3*).

According to their ability to cause human disease, *Leptospira* bacteria were originally divided into fully pathogenic (P1), intermediate pathogenic (P2), and saprophytic or nonpathogenic (S1 and S2) subclades; this phylogenetic separation is confirmed by genome sequencing (*4*,*5*). The pathogenic species, most commonly *L. interrogans*, can cause leptospirosis and Weil’s disease, but the role of intermediate species in human illness is unclear (*5*). Intermediate *Leptospira* spp. have been discovered by environmental sampling of soil and water (*5*), but they have also been found in animals and humans, where they are thought to cause only mild, self-limited illness without liver, kidney, or pulmonary involvement (*5*).

*Leptospira* infections are classically diagnosed by using the microscopic agglutination test (MAT) to detect *Leptospira*-specific antibodies, but diagnosis often requires comparing titers of acute and convalescent serum samples. Culturing *Leptospira* for a definitive bacteriologic diagnosis is difficult and takes weeks to months. Therefore, *Leptospira* bacteria are usually detected by PCR of blood or urine samples and identified by sequencing the amplified genes and comparing those sequences to known *Leptospira* spp. (*6*).

Venezuela is considered a moderate-incidence country for leptospirosis (*7*), but the true incidence is unknown because of a lack of clinical recognition of the disease and difficulties in laboratory diagnosis. To determine the presence of *Leptospira* spp*.*, identify local strains, and evaluate leptospirosis incidence in Venezuela, we performed a prospective study in La Guaira, a small state on Venezuela’s Caribbean coast. Although the study was conducted in 2010–2011 and reporting delayed because of Venezuela’s economic situation, we believe the clinical leptospirosis data and epigenomic study of an intermediate *Leptospira* sp. outbreak remain relevant.

## Methods

### Ethics Approval

The Bioethics Commission of the Instituto Venezolano de Investigaciones Científicas, Caracas, Venezuela, approved the human study. The National Office of Biologic Diversity within the Venezuela Ministry for the Environment (Document 0264) and the Instituto Venezolano de Investigaciones Científicas Commission on Animal Bioethics approved the capture of rodents. 

### Study Area

We included patients with possible leptospirosis in La Guaira State, located on the northern Caribbean coast of Venezuela. La Guaira contains a shipping port and the nation’s principal airport and has a population of ≈353,000. It is a beach resort for residents of Caracas but also contains low socioeconomic urban and rural areas. In the 1999 Vargas tragedy, mudslides destroyed much of the infrastructure of La Guaira (formerly Vargas State), causing thousands of fatalities.

### Patient Selection

We visited Dr. José María Vargas Hospital during March 2010–March 2011 and Dr. Rafael Medina Jiménez Hospital during March–July 2010 and February–March 2011; visits were >2 times per week each. We reviewed diagnoses of new patients at admission and questioned hospital staff about new patients who had clinical symptoms suggestive of leptospirosis. Inclusion criteria were residence or place of work in La Guaira and an initial evaluation that included >1 sign or symptom of leptospirosis as described by the World Health Organization (*8*): fever >38°C with unknown etiology for <21 days, fever with renal failure (anuria, oligouria, or elevated creatinine), abdominal or muscle pain, icterus, conjunctival suffusion, hypokalemia or hyponatremia, hemoptysis or pulmonary hemorrhage, or an initial diagnosis of hepatitis or dengue. After patients voluntarily signed an informed consent form, we interviewed those patients and collected their clinical histories and places of residence. We also consulted the physician’s notes. We excluded patients who were unable to complete the interview or provide adequate data. We enrolled a total of 71 patients from whom blood and urine specimens were obtained. Of those 71 patients, 38 had serologic tests for dengue and 39 for hepatitis A or B. Frozen serum samples from some patients were subsequently tested for hepatitis viruses A and B by PCR (Appendix Table 1). 

### *Leptospira* Cultures

*Leptospira* were cultured at 28–30°C in liquid or semisolid Ellinghausen-McCullough-Johnson-Harris (EMJH) medium with 10% supplement and 50–100 μg/mL of 5-fluorouracil for initial cultures (Appendix). All solutions and media were prepared according to the World Health Organization technical manual (*8*).

### Rodent Capture

We set up Sherman aluminum traps in urban areas close to the residences of patients who were PCR positive for *Leptospira* (Appendix). The species of captured rodents were determined by amplifying and sequencing a subunit of the cytochrome c oxidase gene (*9*).

### Cow Samples

We collected blood with and without EDTA anticoagulant from the caudal vein of 16 crossbred *Bos taurus* × *Bos indicus* (predominantly *Bos taurus*) dairy cows. Cows were 3–10 years of age and located on a farm in Miranda State, Venezuela, ≈30 km from La Guaira State (Appendix). We collected urine samples from the same cows after intramuscular injection of the diuretic furosemide (1 mg/kg). We cultured blood and urine samples and performed PCR for the *Leptospira* genes *rrs* (16S rDNA) and *lipL32*.

### Passaging of Isolates in Hamsters

We intraperitoneally injected *Leptospira* isolates from second to fourth passages of liquid culture into 4-week-old male Syrian golden hamsters (*Mesocricetus auratus*). Sixteen days after injection, we euthanized the hamsters and removed and macerated the kidneys. We placed the kidney tissue into EMJH medium to sediment and then inoculated culture medium with the supernatant.

### Molecular Detection of *Leptospira*

We amplified *lipL32* (*10*) and regions V3–V6 of the *rrs* gene from isolated DNA by using PCR (*11*) (Appendix Table 1). We purified the *rrs* gene amplification products (QIAGEN, https://www.qiagen.com), which were then sequenced by Macrogen (https://www.macrogen.com). We also sequenced the *lig* gene from a few specimens (*12*). We used the *L. interrogans* genes *pntA*, *sucA*, *pfkB*, *tpiA*, *mreA*, *glmU*, and *caiB* (*13*,*14*) for multilocus sequence typing (MLST). We performed variable-number tandem-repeat (VNTR) analysis of *L. interrogans* isolates as previously described (*15*).

### MAT of Bovine Serum Samples

MATs were performed in the bacteriology laboratory of the Instituto Nacional de Investigaciones Agricolas according to 2003 Pan American Health Organization standards (https://www.paho.org/es/documentos/leptospirosis-humana-guia-para-diagnostico-vigilancia-control). MATs were considered positive when >50% of *Leptospira* bacteria were agglutinated.

### Phylogenetic Reconstruction of *L. venezuelensis* Isolates

We used Velvet (*16*) for de novo assembly of genome contigs from sequencing reads of *L. venezuelensis* isolates. We used cow isolate 201502610 (GenBank Biosample accession no. SAMEA5168082) as a reference to map reads from the other *L. venezuelensis* isolates (Appendix).

### Statistics

We performed statistical analyses of patient signs and symptoms and clinical test values by using Stata 13 (StataCorp LLC, https://www.stata.com). We did not adjust p values for multiple statistical testing.

## Results

### PCR and Cultures of Patient Specimens

Through twice-weekly visits to the 2 hospitals in La Guaira state over a 1-year period, we identified 71 patients who met the inclusion criteria (Appendix Tables 2, 3). We PCR amplified the *Leptospira rrs* gene from blood samples of 17, urine samples of 22, and both blood and urine samples of 2 patients. We also cultured *Leptospira* bacteria from blood samples from 13, urine samples from 20, and both blood and urine samples from 3 patients (Appendix Table 4). Using PCR amplification of *Leptospira rrs* in either blood or urine samples as confirmation of leptospirosis, the sensitivity of the *rrs* gene for diagnosing leptospirosis was 46% for blood and 59% for urine specimens; both sample types had 100% specificity. For *lipL32* PCR amplification, sensitivity was 41% for blood, 35% for urine, and 70% when both blood and urine samples were tested; all samples had 100% specificity. For blood cultures, sensitivity was 43%, and specificity was 100%; for urine cultures, sensitivity was 59%, and specificity was 97%; for either positive blood or urine cultures, sensitivity was 95%, and specificity was 97% (Table 1; Appendix Tables 4–6). The *rrs* gene was amplified from 2 patients who had negative *Leptospira* cultures: from a blood specimen of a patient with jaundice and from the urine of a patient who died of severe pulmonary disease.

**Table 1:** Tests used for leptospirosis diagnoses in study of outbreak of intermediate species *Leptospira venezuelensis* spread by rodents to cows and humans in *L. interrogans*–endemic region, Venezuela*

Diagnostic test†	*rrs* PCR+, n = 37‡	*rrs* PCR–, n = 34‡	% Sensitivity (95% CI)	% Specificity (95% CI)	% PPV (95% CI)	% NPV (95% CI)	% Accuracy (95% CI)
*rrs* PCR							
Blood, +	17	0	46 (30–63)	100 (90–100)	100 (80–100)	63 (56–70)	72 (60–82)
Blood, –	20	34	NA	NA	NA	NA	NA
Urine, +	22	0	59 (42–75)	100 (90–100)	100 (85–100)	69 (61–77)	79 (68–88)
Urine, *–*	15	34	NA	NA	NA	NA	NA
Both, +	2	0	NA	NA	NA	NA	NA
Culture							
Blood, +	16	0	43 (27–61)	100 (90–100)	100 (80–100)	62 (55–68)	70 (58–81)
Urine, +	22	1	59 (42–75)	97 (85–100)	96 (76–99)	69 (60–77)	77 (66–87)
Either, +	35	1	95 (82–99)	97 (85–100)	97 (84–100)	94 (81–98)	96 (88–99)
*lipL32* PCR							
Blood, +	15	0	41 (25–58)	100 (90–100)	100 (78–100)	61 (54–67)	69 (57–79)
Urine, +	13	0	35 (20–53)	100 (90–100)	100 (75–100)	59 (53–64)	66 (54–77)
Either, +	26	0	70 (53–84)	100 (90–100)	100 (87–100)	76 (65–84)	85 (74–92)

*Diagnostic values were obtained for *Leptospira* cultures and PCR of *Leptospira* *lipL32* and *Leptospira rrs* (16S rDNA) genes of blood and urine specimens from hospitalized patients. NA, not applicable; NPV, negative predictive value; PPV, positive predictive value; –, negative; +, positive. †Test results are shown for patients who had *Leptospira* detected in blood, urine, or either blood or urine. ‡Total numbers of study patients who had the *Leptospira* *rrs* gene detected by PCR of either blood or urine samples.

Initial diagnoses were similar for patients in this study who had positive or negative *Leptospira* PCR and were most commonly dengue, hepatitis, icteric hemorrhagic syndrome, febrile syndrome, or unknown. Leptospirosis was listed as an initial diagnosis for 6 patients from whom *Leptospira* spp. were isolated and for 1 patient who had negative *Leptospira* cultures. Dengue was diagnosed in 3 patients and hepatitis in 4 patients who had positive *Leptospira* cultures; dengue was diagnosed in 5 patients and hepatitis in 4 patients who had negative cultures. *Leptospira* isolation was not correlated with seasonal variation in precipitation.

We compared PCR sequences of *rrs* with GenBank sequences by using BLAST (*17*). We identified 29 sequences as *L. interrogans*, 3 as *L. noguchii*, and 4 were 99% identical to the intermediate species *L. licerasiae* and *L. wolffi* (Appendix Tables 7, 8); genome sequencing showed those 4 isolates belonged to a novel intermediate species that we then named *L. venezuelensis* (*18*). The *lig* gene (*12*) was amplified by PCR from the urine of the culture-negative patient who died of pulmonary disease and was identified as belonging to *L. interrogans* by using BLAST. Patients who had positive tests for hepatitis or dengue and positive *Leptospira* blood or urine cultures all grew *L. interrogans* and were assumed to be co-infected. Serum samples from *L. venezuelensis*–positive patients were negative for hepatitis viruses A and B (Appendix Table 9).

### Clinical Characteristics

**Figure 1:**
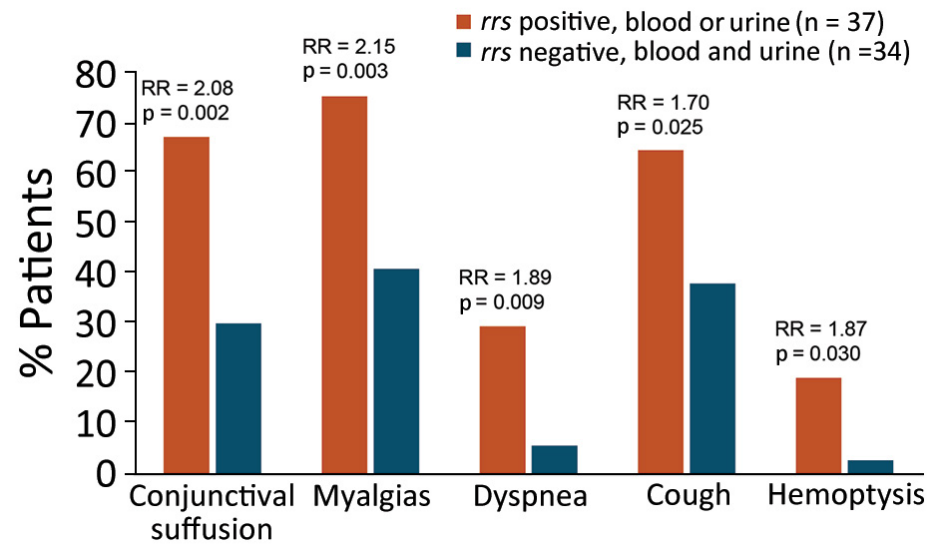
Distinguishing clinical features of hospitalized patients in study of outbreak of intermediate species *Leptospira venezuelensis* spread by rodents to cows and humans in *L. interrogans*–endemic region, Venezuela. The most statistically different clinical symptoms are shown for hospitalized patients considered to have leptospirosis according to positive PCR for the *Leptospria rrs* gene in either blood or urine specimens compared with those without leptospirosis according to negative *rrs* PCR in both blood and urine samples. PCR primers for *rrs* amplify a region of the gene encoding 16S rRNA that is highly conserved in *Leptospira* (Appendix Table 1). One patient whose urine culture grew *L. venezuelensis* was *rrs* PCR negative, and leptospirosis was not diagnosed (Appendix Table 9). Comparisons of all clinical features with 95% CIs were also determined (Appendix Tables 10, 11). RRs and Pearson χ^2^ test p values were calculated by using Stata 13 (StataCorp LLC, https://www.stata.com). RR, risk ratio.

**Figure 2:**
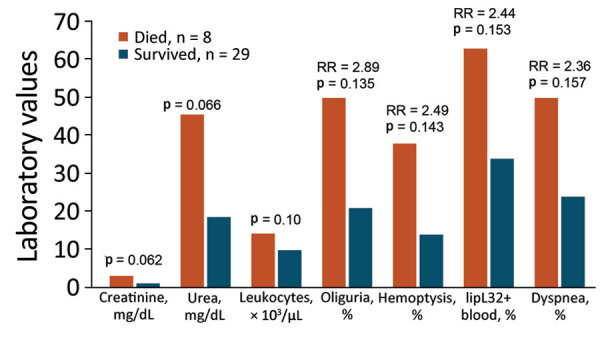
Clinical features most strongly associated with fatal outcomes in study of outbreak of intermediate species *Leptospira venezuelensis* spread by rodents to cows and humans in *L. interrogans*–endemic region, Venezuela. Clinical features are shown for hospitalized patients who had positive PCR tests for the *Leptospira rrs* (16S rDNA) gene in blood or urine and either survived or succumbed to their illness. Laboratory units of measure are indicated on the x axis for each bar. Comparisons of all clinical features with 95% CIs were also determined (Appendix Tables 12, 13). p values comparing creatinine, urea, and number of lymphocytes were obtained from Pearson χ^2^ tests. p values comparing percentages of patients with oliguria, hemoptysis, *lipL32*, and dyspnea were obtained from 2-tailed *t*-tests. All statistical calculations were performed by using Stata 13 (StataCorp LLC, https://www.stata.com). RR, risk ratio.

Patients who had PCR-amplified *rrs* were more likely to have conjunctival suffusion, dyspnea, cough, hemoptysis, and myalgias (Figure 1; Appendix Tables 10, 11). Eight (27%) of the 30 patients who had *L. interrogans* infections died of their illness, whereas no deaths were recorded among the 34 patients who had no evidence of leptospirosis. Leptospirosis patients who died had more severe infections, with pulmonary and renal involvement, than did those who survived (Figure 2; Appendix Tables 12, 13). Patients who died of leptospirosis had more hemoptysis but less abdominal pain and myalgias and also had higher mean urea and creatinine levels, higher leukocyte counts, higher percentages of neutrophils, and lower percentages of lymphocytes than those who survived.

### Cultures from Captured Rodents

To delineate reservoir hosts for *Leptospira*, we captured 45 rodents from 27 communities where patients who had positive cultures resided. We captured 30 *Mus musculus* mice and 11 *Rattus rattus* and 4 *R. norvegicus* rats. We amplified the *rrs* gene by PCR and cultured *Leptospira* from kidney tissue samples from all 45 rodents; 36 (80%) isolates were *L*. *interrogans*, 4 (9%) were *L. noguchii*, 3 (7%) were the intermediate species *L. fainei*, and 2 (4%) were *L. venezuelensis*. *L. interrogans* was isolated from all 3 rodent species, *L. noguchii* was isolated only from mice, and the intermediate species *L. fainei* and *L. venezuelensis* were only isolated from *R*. *rattus* rats.

### *Leptospira* in Cows on Nearby Farm

*Leptospira* spp. are known to infect cattle. In a preliminary study, we performed MATs on serum samples from 48 cows on 8 small farms in adjacent Miranda State. We found *Leptospira*-specific antibodies against >1 *Leptospira* serovars in 2 animals from a single farm located ≈30 km from where the leptospirosis patients in this study resided. We then obtained blood and urine specimens from 16 cows randomly selected from that single farm and performed MATs against live antigens of 23 *Leptospira* reference strains; 9 samples agglutinated >1 serovar (Table 2; Appendix Table 14). Of those 9 cows, 8 had urine positive for *Leptospira* *rrs* by PCR; 2 urine samples had positive cultures of *L. interrogans*, and 7 had positive cultures of *L. venezuelensis.* The cows that had *L. venezuelensis*–positive urine had MAT titers of 1:400 to 1:800 against reference strain *L. interrogans* serovar Wolffi, serogroup Sejroe. The 2 *L. interrogans*–positive cows had high MAT titers for other serovars: 1:400 for *L. hebdomadis* (cow 5) and 1:400 for *L. mini* (cow 9). Cows 1 and 8 were negative according to MATs and *rrs* PCR of their urine, but their blood samples were *rrs* PCR–positive. Urine of cow 8 grew *L. interrogans*, whereas urine of cow 1 grew *L. venezuelensis* (Table 2; Appendix Table 14). Cow 11 had a MAT titer of 1:1,600 for *L. interrogans* serovar Bataviae and *rrs*-positive urine, but no *Leptospira* spp. were isolated from either the blood or urine. *L. venezuelensis* isolates did not agglutinate with antiserum to common *L. interrogans* serovars, although antiserum to serovar Wolffi was not included.

**Table 2:** Analysis of blood and urine specimens from cows in study of outbreak of intermediate species *Leptospira venezuelensis* spread by rodents to cows and humans in *L. interrogans*–endemic region, Venezuela*

Cow no.	*rrs* PCR			*lipL32* PCR			Serology			Cultures		Sequenced species†
	Blood	Urine		Blood	Urine		MAT titer	Serovar		Blood	Urine	
1	+	–		–	–		Negative	NA		+	–	*L. venezuelensis*
2	–	–		–	–		Negative	NA		–	–	NA
3	–	+		–	–		1:800	*L. wolffii*		–	+	*L. venezuelensis*
4	–	–		–	–		Negative	NA		–	–	NA
5	–	+		–	+		1:400	*L. hebdomadis*		–	+	*L. interrogans*
6	–	–		–	–		Negative	NA		–	–	NA
7	–	+		–	–		1:800	*L. wolffii*		–	+	*L. venezuelensis*
8	+	–		+	–		Negative	NA		+	–	*L. interrogans*
9	–	+		–	+		1:400	*L. mini*		–	+	*L. interrogans*
10	–	–		–	–		Negative	NA		–	–	NA
11	–	+		–	–		1:1,600	*L. bataviae*		–	–	NA
12	–	+		–	–		1:800	*L. wolffii*		–	+	*L. venezuelensis*
13	–	+		–	–		1:400	*L. wolffii*		–	+	*L. venezuelensis*
14	–	+		–	–		1:800	*L. wolffii*		–	+	*L. venezuelensis*
15	–	–		–	–		Negative	NA		–	–	NA
16	–	+		–	–		1:400	*L. wolffii*		–	+	*L. venezuelensis*

*Results for MAT serology, cultures, and PCR of the *Leptospira* *lipL32* and *rrs* (16S rDNA) genes from cultured isolates. MAT, microscopic agglutination test; NA, not applicable; –, negative; +, positive. †*Leptospira* spp. were determined by sequencing the PCR-amplified *rrs* gene.

### Growth in Hamsters

We purified all *Leptospira* isolates by injecting early passage cultures into the peritoneal cavities of Syrian golden hamsters and performing necropsies 16 days after inoculation; 3 hamsters infected with *L. interrogans* and 1 infected with *L. noguchii* died before 16 days. We cultured aliquots of macerated kidney extracts from all inoculated hamsters and performed PCR to detect *Leptospira* *rrs*. In each case, sequences of *rrs* from the hamster kidney extracts were identical to the sequences from the corresponding original specimens and also the *Leptospira* cultured from those hamster kidney extracts.

### Molecular Epidemiology of *L. interrogans*

Among the *L. interrogans* strains isolated from humans or rodents, 3 clusters had 7/7 identical MLST alleles; in each cluster, 2 patients resided in the same residential zone (Table 3). Of the 27 different MLST profiles, only 4 were present in the *Leptospira* PubMLST database (https://pubmlst.org/organisms/leptospira-spp), 2 of which (sequence types 27 and 50) were in clusters that had 7/7 identical alleles. Sequence types 20 and 37 were clustered with strains that had 6/7 identical alleles. VNTR clustering was not concordant with MLST clustering (Appendix Table 15). Two of the 3 *L. interrogans* strains isolated from cows had identical alleles in 4 VNTR loci (Appendix Table 16) but were not analyzed by using MLST.

**Table 3:** MLST of *Leptospira interrogans* isolates in study of outbreak of intermediate species *L. venezuelensis* spread by rodents to cows and humans in *L. interrogans*–endemic region, Venezuela*

Isolates†	MLST allele nos.							ST‡
	*glmU*	*pntA*	*sucA*	*tpiA*	*pfkB*	*mreA*	*caiB*	
Human								
CAB-H41	1	1	2	1	7	7	8	NP*
CAY-U48	1	1	2	1	7	4	3	20
CAB-U03	1	1	2	2	7	4	3	NP
MAC-H04	1	1	2	2	7	4	5	NP
** URI-U06**	**1**	**1**	**3**	**2**	**7**	**4**	**3**	NP
** URI-H01**	**1**	**1**	**3**	**2**	**7**	**4**	**3**	NP
CLM-H09	1	1	3	2	4	7	5	NP
CLM-U30	1	3	2	2	4	4	19	NP
MAC-H63	1	3	2	2	7	7	19	NP
CAY-H65	1	3	3	1	4	5	5	NP
SOB-U13	1	12	3	3	10	4	5	NP
MAQ-U18	1	12	3	3	10	5	19	NP
CLM-U22	1	12	2	3	10	6	19	NP
** CLM-U28**	**1**	**12**	**3**	**3**	**10**	**6**	**19**	27
** CLM-H08**	**1**	**12**	**3**	**3**	**10**	**6**	**19**	27
** GUA-H40**	**1**	**12**	**3**	**3**	**10**	**6**	**19**	27
** CLM-U45**	**3**	**3**	**3**	**2**	**4**	**5**	**5**	NP
** CLM-U47**	**3**	**3**	**3**	**3**	**4**	**5**	**5**	37
NAG-U02	6	1	3	2	4	7	3	NP
CAY-U49	6	1	3	3	76	7	3	NP
CLM-U46	6	2	3	3	7	7	19	NP
CLM-U24	6	1	3	12	4	5	5	NP
GUA-H52	6	3	2	2	4	4	3	NP
GUA-H64	6	3	2	3	4	7	5	NP
CAB-U11	6	3	3	2	4	5	5	NP
GUA-H21	6	3	3	3	1	7	5	NP
CAO-U23	6	3	3	3	4	5	19	NP
** MAQ-H53**	**6**	**8**	**2**	**2**	**9**	**7**	**5**	50
** MAQ-H60**	**6**	**8**	**2**	**2**	**9**	**7**	**5**	50
Rat								
CLM-R09-A	1	1	2	2	7	4	8	NP
CLM-R11-A	1	1	3	3	4	6	19	NP
**SOB-R13-B§**	1	12	3	3	10	6	19	27

*Bold font indicates isolates that had identical profiles. MLST, multilocus sequence typing; NP, not present in database; ST, sequence type. †The first 3 letters for each isolate indicate the area of the patient’s residence or where the rodent was captured in the state of La Guaira, Venezuela: CAB, Caraballeda; CAO, Caruao; CAY, Carayaca; CLM, Catia La Mar; GUA, La Guaira; MAC, Macuto; MAQ, Maiquetia; NAG, Niguata; SOB, Soublette; or URI, Urimare. The fourth letter is H (isolated from human blood), U (isolated from human urine), or R (isolated from rat tissue). ‡STs found in the *Leptospira* PubMLST database (https://pubmlst.org/organisms/leptospira-spp) (*14*). §Rat sequence shared an MLST profile for some alleles with human isolates CLM-U28, CLM-H08, and GUA-H40.

### New Intermediate Species of *Leptospira*

*L. venezuelensis*, isolated from 4 patients (Appendix Table 9), 2 rodents, and 7 cows, is located on the phylogenetic tree within the *Leptospira* intermediate pathogen or P2 subclade (*5*). Three of the 4 patients infected with *L. venezuelensis* resided in the same municipality; the fourth patient resided in an adjacent district. This municipality was the most frequent residence of leptospirosis patients, home to 10 of 32 patients with other *Leptospira* infections. Of the 2 rats infected with *L. venezuelensis*, 1 was trapped in the same municipality and the other in a nearby district.

**Figure 3:**
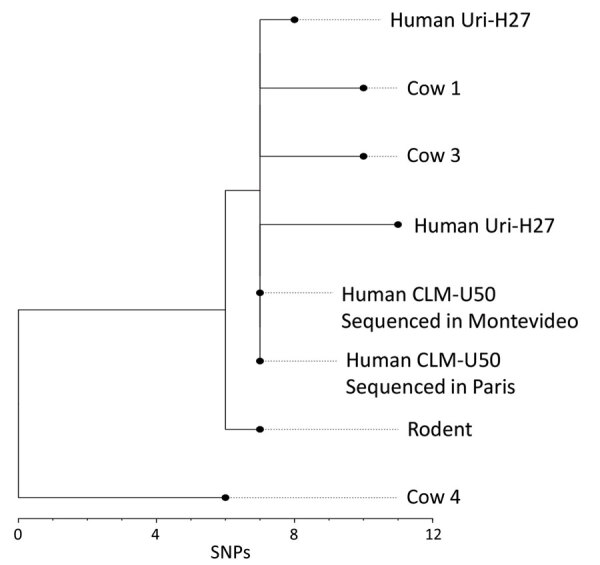
Phylogenetic analysis of *Leptospira venezuelensis* isolates in study of outbreak of intermediate species *L. venezuelensis* spread by rodents to cows and humans in *L. interrogans*–endemic region, Venezuela. Branch length indicates the number of SNPs separating *L. venezuelensis* strains. Phylogenetic tree was reconstructed according to comparisons of whole-genome sequences from 6 *L. venezuelensis* strains isolated from hospitalized leptospirosis patients in La Guaira State on the Caribbean coast of Venezuela, from rodents captured near the residences of hospitalized leptospirosis patients, and from dairy cows on a farm 30 km away from La Guaira State. Human isolate CLM-50 was sequenced at both the Institute Pasteur in Paris, France, and the Institute Pasteur in Montevideo, Uruguay. Human isolate Uri-H27 was sequenced twice at the Institute Pasteur in Paris; the genome of the isolate after many passages in culture contained 3 SNPs that were not present in the same isolate from an earlier passage. Scale bar indicates number of SNPs per site. SNP, single-nucleotide polymorphism.

Phylogenetic reconstruction of genomes from the 6 sequenced *L. venezuelensis* isolates uncovered limited genetic diversity (Figure 3). The isolates from human, rodent, and bovine hosts all differed by <12 single-nucleotide polymorphisms (SNPs), suggesting a recent outbreak of a *L. venezuelensis* strain that was spread, presumably by rodents, to different host populations. The *L. venezuelensis* genome sequence data have been deposited in GenBank (Biosample accession nos. SAMEA5168082, SAMEA5168083, SAMEA5168130, SAMEA5168133, SAMEA5168318, SAMN06855518, SAMN39993761, SAMN39993762, and SAMN39993763).

## Discussion

The true incidence of leptospirosis in La Guaira state has been unknown, likely because it has been difficult or impossible to diagnose and has not been considered by clinicians, even in patients with characteristic signs and symptoms. Our prospective search for leptospirosis cases in La Guaira’s 2 hospitals during a 1-year period found *rrs* PCR evidence of *Leptospira* spp. in blood or urine specimens from 37 hospitalized patients, including 8 patients who died. We also cultured *Leptospira* from 36 patient samples. Two patients with positive *rrs* PCR had negative cultures, but 1 of those patients had an *L. interrogans lig* gene fragment amplified from their urine. The population of La Guaira is ≈353,000, corresponding to a borderline high incidence of 10 leptospirosis cases/100,000 population. However, this figure is almost certainly an underestimate because the study only included patients ill enough to require hospitalization and did not capture patients with less severe illness, who represent up to 90% of leptospirosis cases (*2*).

*Leptospira* spp. were isolated from the kidneys of all 45 rodents captured in the region. *Leptospira* species distributions were similar in rodents and humans; most isolates were *L. interrogans,* which is globally the species most associated with severe human illness. *L. venezuelensis* was also isolated from 7 cows on a nearby farm, whereas *L. interrogans* was isolated from only 3 cows on the same farm (Table 2; Appendix Table 14).

Leptospirosis is difficult to diagnose in a clinically useful time frame, but *rrs* PCR of both blood and urine samples detected 37 cases. The most discriminative clinical finding in patients was conjunctival suffusion (*19*), but *Leptospira*-positive patients also had more myalgias, dyspnea, cough, and hemoptysis than did hospitalized *Leptospira*-negative patients. *L. interrogans* was recovered from patients with the most severe cases, and 27% (8/30) of *L. interrogans*–infected patients died. However, for patients with mild to moderate disease, the infecting species could not be distinguished by patient signs, symptoms, or laboratory values (Appendix Table 8).

The intermediate species *L. fainei* was isolated from the kidneys of 3/45 captured rodents. *L. fainei* has been reported to cause disease in humans (*20*) but was not isolated from any human patient or bovid in this study. *L. venezuelensis* is phylogenetically closer to other intermediate species reported to cause human illness, such as *L. liceraciae* (*21*) and *L. wolffi* (*22*) and is phylogenetically close to *Leptospira* spp. isolated from environmental samples in Malaysia, Mayotte, and New Caledonia (*5*).

Few studies have been conducted to determine the phylogenetic relatedness of different strains of *Leptospira* spp. isolated from a particular geographic region. *L. interrogans* isolates from this study had many MLST profiles, including clusters of profiles found in the *Leptospira* PubMLST database (Table 3). MATs showed that serum samples from 3 cows each reacted to a different *L. interrogans* serovar, including 2 whose isolates had the same VNTR pattern (Appendix Tables 14, 16). The heterogeneity of *L. interrogans* strains suggests a long-term endemic presence in the local rodent population. In contrast, the genomes of 6 *L. venezuelensis* isolates differed by a maximum of 11 SNPs (Figure 3), suggesting an outbreak strain. Although only 6 of the 13 *L. venezuelensis* isolates were sequenced, they were obtained from a diverse sampling of hospitalized humans, rats captured in La Guaira, and cows on a farm 30 km away from patient residences. Unless *L. venezuelensis* has a mutation rate even slower than slow-mutating *Mycobacterium tuberculosis* (*23*), the low genetic diversity reflects a recently expanded bacteria population. Greater genomic heterogeneity would be expected if *L. venezuelensis* evolved from a local environmental *Leptospira* sp. Instead, the close genomic similarity between isolates suggests a recent introduction of *L. venezuelensis* into the region, perhaps arriving with rats on a ship that docked in the port of La Guaira and then spread within the local rodent population.

Infections with intermediate clade *Leptospira* spp. have only rarely been associated with icteric human illness (*6*), but 3 of 4 patients from whom *L. venezuelensis* was isolated were icteric, had liver aminotransferase values >250 (Appendix Table 9) and negative test results for hepatitis viruses A and B. Only 1 patient with *L. venezuelensis* infection was tested for dengue, but all 4 had platelet levels within reference ranges, which is uncharacteristic for acute dengue.

Although intermediate *Leptospira* spp. are thought to be incapable of surviving in an animal model, infection of rats has been reported for the intermediate species *L. licerasiae* (*24*). We recovered all 13 *L. venezuelensis* isolates from hamster kidneys 16 days after intraperitoneal inoculation of low passage isolates, although later passages of the same isolates could not be recovered from hamsters after high-dose intraperitoneal infections (data not shown). The acquisition of SNPs and loss of virulence during *in vitro* passages of *Leptospira* isolates has been previously described (*25*,*26*).

*Leptospira* intermediate species are often isolated from environmental samples (*5*), but it seems unlikely that *L. venezuelensis* was an environmental or laboratory contaminant. The *rrs* PCR of the original human, bovine, and rodent specimens; the isolate cultures; and hamster infection studies were all performed separately before sequencing results were available, and the samples containing *L. venezuelensis* were not temporally linked. The MAT titers of serum samples from *L. venezuelensis*–positive bovids all showed the same presumed cross-reaction with *L. interrogans* serovar Wolffi, consistent with the genomic evidence of an outbreak strain. Human disease causality could be confirmed by high or rising MAT titers in patient serum samples, but acute serum samples from 2 *L. venezuelensis* and 4 *L. interrogans* patients did not have titers >1:50, and convalescent patient blood samples were not collected.

In Argentina (*27*), *L. wolffii* was isolated from a patient who died of a severe respiratory syndrome, but PCR results suggested an *L. interrogans* co-infection. Similarly, 2 of the 4 *L. venezuelensis*–positive patients in this study had positive *lipL32* PCR results (Appendix Table 9). The *lipL32* primers were designed to amplify *lipL32* from *L. interrogans* or other pathogenic *Leptospira* spp. but not from intermediate species, such as *L. venezuelensis*. Although the amplified *lipL32* fragments were not sequenced, the 2 *lipL3-*positive patients could have been co-infected with *L. venezuelensis* and *L. interrogans.* Another patient from whom *L. venezuelensis* was cultured had negative *rrs* PCR results in both blood and urine. The pathogenicity of this intermediate species could not be confidently evaluated from the 4 *L. venezuelensis*–positive patients in this study.

In conclusion, an *L. venezuelensis* outbreak circulating in rodents appears to have spread to cows in the region and also infected humans, in whom it might have caused febrile illness with hepatic involvement. Our findings indicate the need for increased awareness of leptospirosis prevalence and characteristics in Venezuela and other tropical, rodent infested coastal regions and also indicates an urgent need for rapid point-of-care tests to diagnose leptospirosis and improve patient treatment and outcomes.

Appendix Additional information for outbreak of intermediate species *Leptospira venezuelensis* spread by rodents to cows and humans in *L. interrogans*–endemic region, Venezuela.
