# Correction: Vol. 30, No. 8

**DOI:** 10.3201/eid3009.C13009

**Published:** 2024-09

**Keywords:** errata, erratum, correction

The name of author Carlos E. Sanz-Rodriguez was misspelled in Outbreak of Intermediate Species *Leptospira venezuelensis* Spread by Rodents to Cows and Humans in *L. interrogans*–Endemic Region, Venezuela (L. Caraballo et al.). The article has been corrected online (https://wwwnc.cdc.gov/eid/article/30/8/23-1562_article).

