# Supplementary material for: Outbreak of Intermediate Species Leptospira venezuelensis Spread by Rodents to Cows and Humans in L. interrogans–Endemic Region, Venezuela
Source: Emerg Infect Dis. 2024 Aug;30(8):1514–22. doi: 10.3201/eid3008.231562 (PMC11286060; doi:10.3201/eid3008.231562)
Supplement: Appendix — Additional information for outbreak of intermediate species Leptospira venezuelensis spread by rodents to cows and humans in L. interrogans–endemic region, Venezuela. [file 23-1562-Techapp-s1.pdf]

Article DOI: https://doi.org/10.3201/eid3008.231562

EID cannot ensure accessibility for supplementary materials supplied by authors. Readers who have difficulty accessing supplementary content should contact the authors for assistance.

Outbreak of Intermediate Species *Leptospira venezuelensis* Spread by Rodents to Cows and Humans in *L. interrogans*–Endemic Region, Venezuela

Appendix

Additional Methods

Leptospira Cultures

Leptospira were cultured at 28–30°C in liquid or semisolid Ellinghausen-McCullough-Johnson-Harris (EMJH) medium containing 10% supplement (bovine serum albumin, Tween 80, chlorides, sulfides, and vitamins), and 50–100 μg/mL of 5-fluorouracil (5-FU) for initial cultures. All solutions and culture media were prepared according to the World Health Organization technical manual (*1*) using freshly made copper and iron solutions. All culture media, antimicrobial drugs, and solutions were prepared by using analytical or molecular biology grade reagents from commercial sources.

Four or five drops of whole patient blood were inoculated into 5 mL of EMJH 5-FU broth in duplicate. For urine cultures, 500 μL of unconcentrated urine were inoculated into 5 mL of EMJH 5-FU broth. In addition, 5 mL of urine was centrifuged at 3220 × g for 10 minutes, the supernatant was discarded, the pellet was resuspended in 500 μL EMJH 5-FU, and then 500 μL was inoculated into 5 mL EMJH 5-FU broth. When *Leptospira* growth was noted, primary cultures were passaged by using serial dilutions (10^{-1}, 10^{-2}, 10^{-3}) into semisolid EMJH-5-FU medium, which was then monitored for growth by dark field microscopy weekly for several months.

After the exclusive presence of spirochetes typical of *Leptospira* was observed, the culture was passaged into fresh sterile EMJH 5-FU (semisolid medium at 1:10 vol/vol and 2× liquid media at 1:1 vol/vol, both in duplicate) and then monitored weekly for growth by dark field microscopy. After good growth was noted, the strains were passed weekly to fresh semisolid EMJH medium at 1:10 vol/vol until 1,000 bacteria cells/mL was obtained or until the characteristic Dinger's ring was observed, indicating growth of *Leptospira*.

Rodent Capture

The National Office of Biologic Diversity of the Venezuela Ministry for the Environment (Document 0264) and the Instituto Venezolano de Investigaciones Científicas Commission on Animal Bioethics approved the capture of rodents in Sherman aluminum traps set in urban areas close to residences of patients who were PCR positive for *Leptospira*. The species of captured rodents were determined by isolating DNA from liver samples by using a DNeasy Blood and Tissue kit (QIAGEN, https://www.qiagen.com) and then sequencing a PCR-amplified subunit of the cytochrome c oxidase gene (*2*).

The rodents were euthanized according to the 2010 Guidelines of the Canadian Council on Animal Care (https://ccac.ca/en/guidelines-and-policies/the-guidelines). The kidneys were removed by using sterile technique, macerated, placed in a 15 mL tube containing 5 mL of EMJH 5-FU broth, and left to sediment at room temperature for 25 minutes. The supernatants were examined by dark field microscopy for the presence of *Leptospira* and 500 μL of supernatant was inoculated into 2 tubes of EMJH 5-FU broth.

Bovine Samples

Blood from the caudal vein of cows on farms in Miranda State, ≈30 km from La Guaira State, was collected with and without EDTA as an anticoagulant. Two or 3 drops of uncoagulated blood were inoculated onto 5 mL of semisolid EMJH medium in duplicate, incubated at 30°C, and examined weekly by using darkfield microscopy. After spirochetes were detected, the cultures were inoculated into fresh semisolid media and incubated at 30°C.

Urine samples from cows were collected in sterile 50 mL tubes after intramuscular injection of the diuretic furosemide (1 mg/kg). Ten mL of urine samples were centrifuged for 30 minutes at 3,220 × g and then 0.5 mL of the pellet was mixed with 4.5 mL liquid EMJH medium (dilution 1:10) supplemented with albumin and 5-FU and incubated at 30°C. In addition, 10 mL

of urine samples were passed through a 0.45 micron syringe filter and 500 μL of the filtrate was inoculated in duplicate onto semisolid EMJH medium and incubated at 30 C. Two or 3 drops of uncoagulated blood were inoculated onto 5 mL semisolid EMJH medium in duplicate, incubated at 30°C, and examined weekly by using darkfield microscopy. After spirochetes were detected, the cultures were inoculated onto fresh semisolid EMJH medium and incubated at 30°C.

Molecular Detection of Pathogenic *Leptospira* in Blood and Urine

Patient blood samples (2.5 mL) were collected in EDTA tubes and DNA was isolated by using the AxyPrep Blood Genomic DNA Miniprep kit (Corning, https://www.corning.com). Midstream urine samples (15 mL) were collected and adjusted to pH 7.5 with 1–2 mL 8.5% sodium bicarbonate supplemented with 1% 5-FU; DNA was isolated as previously described (*3*). DNA from 300 μL blood collected from cow caudal veins was extracted by using the Geneaid DNA Isolation Kit (Geneaid, https://www.geneaid.com) and stored at −20°C until PCR analysis. Immediately after urine samples were collected from cows, ≈30 mL urine per cow was spun at 1,600 × g for 30 minutes. Then, 200 μL of the pellet was processed by using the Geneaid kit, and the isolated DNA was used in standard PCR with primers for *lipL32* and regions V3–V6 of the *rrs* gene (*4*) (Appendix Table 1). Detection of *lipL32* was conducted as previously described (*5*). The *rrs* gene PCR products were purified and sequenced by Macrogen (https://www.macrogen.com).

Molecular Analysis of Cultured *Leptospira* Species

To identify *Leptospira* spp. in cultured material, the V2–V9 regions of the *rrs* gene (16S rRNA) were PCR amplified by using the primers rrsfull-D and rrsfull-R (Appendix Table 1). The resulting 1,500 bp product was then subjected to a second round of PCR of *rrs* regions V3–V6 by using the nested primers 16S-D(int) and 16S-R(int). The primers and nested-PCR strategy used to detect *lipL32* have been previously described (*5*).

The variable-number tandem-repeats VNTR4, VNTR7, VNTR10, and VNTR-Lb5 loci were amplified by PCR as previously described (*6*); products were analyzed on 2% agarose gels and the sizes of the DNA bands were compared with a 100 bp DNA ladder. The genes amplified and sequenced for multilocus sequence typing were *pntA*, *sucA*, *pfkB*, tpiA, *mreA*, *glmU*, and *caiB* (Appendix Table 1) (*6*). When the yield of genomic DNA was poor, a nested PCR protocol and additional primers were used as previously described (*7*); new primers were designed to

amplify *caiB* from *L. interrogans* and *L. noguchii.* The amplified DNA fragments were purified and sequenced (Macrogen) and aligned with GenBank sequences by using BLAST (https://blast.ncbi.nlm.nih.gov); sequences were compared with *Leptospira* reference species obtained from Genbank by using the ClustalW algorithm in MacVector (https://www.macvector.com).

MATs of Cow Serum Samples

Blood without anticoagulant was centrifuged for 10 minutes at room temperature at 3,220 × g. The serum samples were then transferred to new tubes and stored at −20°C until processed at the Bacteriological Animal Health Laboratory at the Instituto Nacional de Investigaciones Agrícolas in Maracay, Aragua, Venezuela. The microscopic agglutination test (MAT) was performed in the Animal Health Laboratory according to the 2001 Pan American Health Organization standards. The serum samples were inactivated in a water bath at 56°C for 30 minutes and, during the screening phase, diluted 1:25 by adding 0.25 mL serum sample to 6 mL phosphate-buffered saline (PBS).

For live antigens, 23 serovars of *Leptospira* were grown in liquid medium, examined by microscopy to rule out contamination, then diluted in PBS to a 0.5 McFarland turbidity standard. MAT assays were performed in 96-well plates with 100 μL of the diluted serum sample and 100 μL of live antigen. A human serum sample known to be negative for *Leptospira* antibodies was used as a negative control, and a well containing equal parts antigen and PBS without serum sample was used as a blank. The plates were incubated at 37°C for 1 hour and then a drop from each well was analyzed for agglutination by using a darkfield microscope with a 16× objective. MAT tests were considered positive when ≥50% of the *Leptospira* were agglutinated. Titering was also performed in 96-well plates by using 2-fold serial dilutions of serum samples in PBS. The titer was the maximum serum sample dilution in which agglutination was observed.

Phylogenetic Reconstruction of *L. venezuelensis* Isolates

Velvet (*8*) was used for de novo assembly of genome contigs obtained from sequencing reads of *L. venezuelensis* isolates. Isolate 201502610 (GenBank Biosample no. SAMEA5168082) was used as a reference to map the sequencing reads from the other *L. venezuelensis* isolates. The 201502610 reference genome contains 29 assembled contigs with a total length of 4,250,319 bp. Sickle (*9*) was used to trim the reads from genome sequencing data

and sequencing reads with a Phred base quality score >20 and read lengths >30 bp were kept for analysis. Sequencing reads were mapped to the reference genome by using Bowtie (*10*), and then SAMtools version 1.3.1 (*11*) was used for calling single-nucleotide polymorphisms that had a mapping quality score >30. Fixed mutations having a frequency of ≥95% and ≥20 supporting reads were identified by using VarScan version 2.3.9 (*12*); small insertions or deletions identified by VarScan were excluded in the analysis. The alignments of polymorphic positions from all *Leptospira* strains were used for phylogenic reconstruction by using MEGA 7.0 (*13*). The neighbor-joining method was used for initial inference of the phylogenic structure under the number of differences model.

Tests for Dengue and Hepatitis

Tests for dengue were performed at the Instituto Nacional de Higiene in Caracas by using a solid phase immunochromatographic assay that detects IgG and IgM against dengue virus serotypes 1–4, together with a rapid chromatographic immunoassay against dengue virus nonstructural protein 1 antigen. Frozen serum samples from *L. venezuelensis*–culture positive patients were tested for hepatitis A virus by using an ELISA to detect virus-specific IgM and tested for hepatitis B by using an ELISA to detect hepatitis B surface antigen and hepatitis B core antibody.

References

1. World Health Organization. Human leptospirosis: guidance for diagnosis, surveillance and control. 2003 [cited 2023 Oct 15]. https://www.who.int/publications/i/item/human-leptospirosis-guidance-for-diagnosis-surveillance-and-control

2. Robins JH, McLenachan PA, Phillips MJ, Craig L, Ross HA, Matisoo-Smith E. Dating of divergences within the *Rattus* genus phylogeny using whole mitochondrial genomes. Mol Phylogenet Evol. 2008;49:460–6. PubMed https://doi.org/10.1016/j.ympev.2008.08.001

3. Boom R, Sol CJ, Salimans MM, Jansen CL, Wertheim-van Dillen PM, van der Noordaa J. Rapid and simple method for purification of nucleic acids. J Clin Microbiol. 1990;28:495–503. PubMed https://doi.org/10.1128/jcm.28.3.495-503.1990

4. Ahmed A, Anthony RM, Hartskeerl RA. A simple and rapid molecular method for *Leptospira* species identification. Infect Genet Evol. 2010;10:955–62. PubMed https://doi.org/10.1016/j.meegid.2010.06.002

5. Bomfim MR, Koury MC. Evaluation of LSSP-PCR for identification of *Leptospira* spp. in urine samples of cattle with clinical suspicion of leptospirosis. Vet Microbiol. 2006;118:278–88. PubMed https://doi.org/10.1016/j.vetmic.2006.07.020

6. Salaün L, Mérien F, Gurianova S, Baranton G, Picardeau M. Application of multilocus variable-number tandem-repeat analysis for molecular typing of the agent of leptospirosis. J Clin Microbiol. 2006;44:3954–62. PubMed https://doi.org/10.1128/JCM.00336-06

7. Agudelo-Flórez P, Murillo VE, Londoño AF, Rodas JD. Histopathological kidney alterations in rats naturally infected with *Leptospira*. Biomedica. 2013;33:82–8. PubMed

8. Zerbino DR. Using the Velvet de novo assembler for short-read sequencing technologies. Curr Protoc Bioinformatics. 2010;11:11.5. PubMed https://doi.org/10.1002/0471250953.bi1105s31

9. Joshi NA, Fass JN. Sickle: a sliding-window, adaptive, quality-based trimming tool for FastQ files. 2011 [cited 2022 Oct 31]. https://github.com/najoshi/sickle

10. Li H, Handsaker B, Wysoker A, Fennell T, Ruan J, Homer N, et al.; 1000 Genome Project Data Processing Subgroup. The sequence alignment/map format and SAMtools. Bioinformatics. 2009;25:2078–9. PubMed https://doi.org/10.1093/bioinformatics/btp352

11. Langmead B, Salzberg SL. Fast gapped-read alignment with Bowtie 2. Nat Methods. 2012;9:357–9. PubMed https://doi.org/10.1038/nmeth.1923

12. Koboldt DC, Zhang Q, Larson DE, Shen D, McLellan MD, Lin L, et al. VarScan 2: somatic mutation and copy number alteration discovery in cancer by exome sequencing. Genome Res. 2012;22:568–76. PubMed https://doi.org/10.1101/gr.129684.111

13. Kumar S, Stecher G, Tamura K. MEGA7: molecular evolutionary genetics analysis version 7.0 for bigger datasets. Mol Biol Evol. 2016;33:1870–4. PubMed https://doi.org/10.1093/molbev/msw054

14. Boonsilp S, Thaipadungpanit J, Amornchai P, Wuthiekanun V, Bailey MS, Holden MTG, et al. A single multilocus sequence typing (MLST) scheme for seven pathogenic *Leptospira* species. PLoS Negl Trop Dis. 2013;7:e1954. PubMed https://doi.org/10.1371/journal.pntd.0001954

15. Thaipadungpanit J, Wuthiekanun V, Chierakul W, Smythe LD, Petkanchanapong W, Limpaiboon R, et al. A dominant clone of *Leptospira interrogans* associated with an outbreak of human leptospirosis in Thailand. PLoS Negl Trop Dis. 2007;1:e56. PubMed https://doi.org/10.1371/journal.pntd.0000056

16. Ahmed N, Devi SM, Valverde Mde L, Vijayachari P, Machang'u RS, Ellis WA, et al. Multilocus sequence typing method for identification and genotypic classification of pathogenic *Leptospira* species. Ann Clin Microbiol Antimicrob. 2006;5:28. PubMed https://doi.org/10.1186/1476-0711-5-28

17. Bomfim MR, Barbosa-Stancioli EF, Koury MC. Detection of pathogenic leptospires in urine from naturally infected cattle by nested PCR. Vet J. 2008;178:251–6. PubMed https://doi.org/10.1016/j.tvjl.2007.07.029

18. Garson JA, Ring C, Tuke P, Tedder RS. Enhanced detection by PCR of hepatitis C virus RNA. Lancet. 1990;336:878–9. PubMed https://doi.org/10.1016/0140-6736(90)92384-T

19. Okamoto H, Okada S, Sugiyama Y, Tanaka T, Sugai Y, Akahane Y, et al. Detection of hepatitis C virus RNA by a two-stage polymerase chain reaction with two pairs of primers deduced from the 5′-noncoding region. Jpn J Exp Med. 1990;60:215–22. PubMed

20. Hu X, Margolis HS, Purcell RH, Ebert J, Robertson BH. Identification of hepatitis B virus indigenous to chimpanzees. Proc Natl Acad Sci USA. 2000;97:1661–4. PubMed https://doi.org/10.1073/pnas.97.4.1661

21. Schaefer S, Glebe D, Wend UC, Oyunbileg J, Gerlich WH. Universal primers for real-time amplification of DNA from all known *Orthohepadnavirus* species. J Clin Virol. 2003;27:30–7. PubMed https://doi.org/10.1016/S1386-6532(02)00108-7

22. Costa-Mattioli M, Cristina J, Romero H, Perez-Bercof R, Casane D, Colina R, et al. Molecular evolution of hepatitis A virus: a new classification based on the complete VP1 protein. J Virol. 2002;76:9516–25. PubMed https://doi.org/10.1128/JVI.76.18.9516-9525.2002

23. Palaniappan RUM, Chang YF, Chang CF, Pan MJ, Yang CW, Harpending P, et al. Evaluation of *lig*-based conventional and real time PCR for the detection of pathogenic leptospires. Mol Cell Probes. 2005;19:111–7. PubMed https://doi.org/10.1016/j.mcp.2004.10.002

Appendix Table 1. PCR primers used in the study

Primer name	Primer sequence, 5′–3′	Annealing temperature, °C	Reference
glmU-D(Ext)	AGGATAAGGTCGCTGTGGTA	52	(*14*)
glmU-R(Ext)	AGTTTTTTTCCGGAGTTTCT		
glmU-D(Int)	GGAAGGGCACCCGTATGAA	52	(*15*)
glmU-R(Int)	TCCCTGAGCGTTTTGATTT		
pntA-D(Ext)	TGCCGATCCTACAACATTA	52	(*15*)
pntA-D(Int)	TAGGAAARATGAAACCRGGAAC		
pntA- R	AAGAAGCAAGATCCACAAYTAC		(*14*)
sucA-D(Ext)	TCATTCCACTTYTAGATACGAT	58	(*14*)
sucA-R(Ext)	TCTTTTTTGAATTTTTGACG		
sucA-D(int)	AGAAGAGGCCGGTTATCATCAG	52	(*15*)
sucA-R(int)	TTCCGGGTCGTCTCCATTTA		
tpiA-D(Ext)	TTGCAGGAAACTGGAAAATGAAT	52	(*14*)
tpiA- R(Ext)	GTTTTACRGAACCHCCGTAGAGAAT		
tpiA-D(int)	AAGCCGTTTTCCTAGCACATTC	52	(*15*)
tpiA-R(int)	AGGCGCCTACAAAAAGACCAGA		
*pfkB*13-D(Ext)	CGGAGAGTTTTATAARAAGGACAT	52	(*14*)
*pfkB*13-R(Ext)	AGAACACCCGCCGCAAAACAAT		
pfkB-D(int)	CCGAAGATAAGGGGCATACC	52	(*15*)
pfkB-R(int)	CAAGCTAAAACCGTGAGTGATT		
*mreA*13-D(Ext)	GGCTCGCTCTYGACGGAAA	58	(*14*)
*mreA*13-R(Ext)	TCCRTAACTCATAAAMGACAAAGG		
mreA-D(int)	GTAAAAGCGGCCAACCTAACAC	52	(*15*)
mreA-R(int)	ACGATCCCAGACGCAAGTAA		
caiB-D(Ext)	TAGAAATTTTGCRGGACACG	54	This study
caiB-R(Ext)	TAAAGTTCGGTAGATAGACT		
caiB(int)	ACACCTCAGATTCCAGGAT	55	
caiB(int)	GGAATACCGGRTCCTTAAT		
*rrs*full-D	GCTCAGAACTAACGCTGGCG	60	This study
*rrs*full-R	TATTCACCGCGGCATGCTGA		
16s-D(int)	CATGCAAGTCAAGCGGAGTA	58	(*16*)
16s-R(int)	AGTTGAGCCCGCAGTTTTC		
lipL32F	ATCTCCGTTGCACTCTTTGC	58	(*16*)
lipL32R	ACCATCATCATCATCGTCCA		
lipl32-P662	CTAAGTTCATACCGTGATTT	58	(*17*)
lipl32 P663	TTCTGACGCGACTAAGTAAT		
lipl32 Internal 1	GACGGTTTAGTCGATGGAAAC	58	(*17*)
lipl32Internal 2	GGGAAAAGCAGACCAACAGA		
209-5′NCR HCV	ATACTCGAGGTGCACGGTCTACGAGACCT	50	(*18*)
211-5′NCR HCV	CACTCTCGAGCACCCTATCAGGCAGT		
939-5′NCR HCV	CTGTGAGGAACTACTGTCTT	53	(*19*)
940-5′NCR HCV	TTCACGCAGAAAGCGTCTAG		
58-Surface HBV	CCTGCTGGTGGCTCCAGTTC	58	(*20*)
1101n-Surface HBV	GAAAGGCCTTGTAAGTTGGCGAG		
S3s-Surface HBV	TGCCTCATCTTCTTRTTGGTTCT	53	(*21*)
S3as-Surface HBV	CCCCAAWACCAVATCATCCAT		
HAV1-VP1 HAV	GTTTTGCTCCTCTTTATCATGCTATG	52	(*22*)
HAV7-VP1 HAV	CTGGAGTGAACCAGGCCATGCCATC		
HAV6-VP1 HAV	AGGAAATGTCTCAGGTACTTTCTTTGCTAAAACTG		
Lig1	TCAATCAAAACAAGGGGCT	48	(*23*)
Lig2	ACTTGCATTGGAAATTGAGAG		

Appendix Table 2. Age of patients in the study according to sex and PCR results for *Leptospira rrs* gene

	rrs-positive blood or urine		*rrs*-negative blood and urine	
Patient sex	No. patients	Average age, y (range)	No. patients	Average age, y (range)
M	21	34.7 (13–81)	22	30.3 (2 mo–72 y)
F	15	31.6 (4–71)	13	36.6 (16–60)
Total	36	33.4	35	32.7

Appendix Table 3. Source hospitals of patients in the study according to sex and PCR results for *Leptospira rrs* gene

	rrs-positive blood or urine			*rrs*-negative blood and urine		
Hospital where patient was contacted	M	F	Total	M	F	Total
Dr. Jose Maria Vargas	20	11	31	18	12	31
Dr. Rafael Medina Jiménez (Pariata)	2	4	6	3	1	4
Total no. patients	22	15	37	21	13	34

Appendix Table 4. PCR results for *Leptospira* spp. compared with cultures of blood and urine samples from hospitalized patients*

					lipL32 PCR						*rrs* PCR					
	Positive culture				Blood		Urine		Both		Blood		Urine		Both	
Leptospira spp.	Blood	Urine	Both	Total	Pos	Neg	Pos	Neg	Pos	Neg	Pos	Neg	Pos	Neg	Pos	Neg
L. interrogans	10	16	3	29	11	18	12	17	2	8	12	17	19	10	2	0
L. noguchii	2	1	0	3	2	1	0	3		1	3		0	3	0	0
L. venezuelensis	1	3	0	4	1	3	1	3		2	1	3	2	2		1
Total	13	20	3	36	14	22	13	23	2	11	16	20	21	15	2	1

*PCR was conducted to detect *lipL32* and *rrs Leptospira* genes. Neg, negative; Pos, positive.

Appendix Table 5. Average number of days from onset of symptoms in hospitalized patients to collection of blood or urine positive for *Leptospira* by PCR or culture*

	Blood		Urine		
Detection method	No. samples	No. days (range)	No. samples	No. days (range)	p value†
lipL32 PCR	15	9.0 (1–21)	13	10.38 (1–21)	0.52
rrs PCR	17	9.18 (1–21)	20	10.55 (1–22)	0.48
Culture	16	9.69 (1–21)	20	10.43 (1–21)	0.35

*PCR was conducted to detect *lipL32* and *rrs Leptospira* genes.
†*t*-test was used to compare duration of symptoms before a positive test for *Leptospira* was obtained in blood verses urine.

Appendix Table 6. Comparison of positive PCR results for *Leptospira* in blood, urine, or both samples from hospitalized patients whose cultured blood or urine specimens grew *Leptospira**

	lipL32-positive PCR			*rrs*-positive PCR		
Leptospira-positive cultures	Blood	Urine	Blood and urine	Blood	Urine	Blood and urine
Blood, n = 13	11	0	0	13	0	0
Urine, n = 20	2	10	1	1	17	1
Blood and urine, n = 3	0	1	1	1	2	0

*PCR was conducted to detect *lipL32* and *rrs Leptospira* genes.

Appendix Table 7. Average number of days of symptoms in hospitalized patients before specimens were obtained for diagnostic tests according to *Leptospira* spp. isolated and positive or negative test results

	lipL32 PCR, blood				*lipL32* PCR, urine				*rrs* PCR, blood				*rrs* PCR, urine				Blood culture				Urine culture			
	Pos		Neg		Pos		Neg		Pos		Neg		Pos		Neg		Pos		Neg		Pos		Neg	
Leptospira spp.	Pts	Days	Pts	Days	Pts	Days	Pts	Days	Pts	Days	Pts	Days	Pts	Days	Pts	Days	Pts	Days	Pts	Days	Pts	Days	Pts	Days
L. interrogans	11	7.82	18	10.44	12	10.58	17	8.65	12	8.75	17	9.94	12	11.42	16	8.06	13	9.85	16	9.13	19	10.37	10	7.7
L. noguchii	2	10.5	1	2	0	NA	3	7.67	3	7.67	0	NA	1	2	2	10.5	2	6.5	1	10	1	10	2	6.5
L. venezuelensis	1	14	3	11	1	8	3	13	1	14	3	11	3	11	1	14	1	14	3	11	3	11	1	14

*PCR was conducted to detect *Leptospira lipL32* and *rrs* genes. Data show number of patients in each group and average number of days. NA, not applicable; Neg, negative; Pos, positive; Pts, patients.

Appendix Table 8. Symptoms or treatments of hospitalized patients from whom the indicated *Leptospira* species was isolated

	No. patients infected with *Leptospira* spp.		
Symptoms/treatments	*L. interrogans*	*L. noguchii*	*L. venezuelensis*
Fever			
Yes	28	3	4
No	1	0	0
Antimicrobial drug treatment			
Yes	16	1	2
No	8	2	1
Icterus			
Yes	20	3	4
No	9	0	0
Conjuntival suffusion			
Yes	19	3	3
No	10	0	1
Dyspnea			
Yes	10	0	1
No	19	3	3
Hemoptysis			
Yes	5	0	1
No	23	3	3
Cough			
Yes	20	0	3
No	9	3	1
Oliguria			
Yes	8	0	2
No	19	3	2
Vomiting			
Yes	12	0	2
No	17	3	2
Diarrhea			
Yes	2	0	0
No	27	3	4
Rash			
Yes	6	0	1
No	23	3	3
Myalgias			
Yes	19	3	4
No	10	0	0
Arthralgias			
Yes	15	2	4
No	12	1	0
Abdominal pain			
Yes	17	3	2
No	12	0	2

Appendix Table 9. Laboratory results for the 4 hospitalized patients from whom *L. venezuelensis* was isolated*

Patient no.	*lipL32* +	*rrs* +	Culture +	AST, IU/L	ALT, IU/L	Indirect bilirubin, mg/dL	Direct bilirubin, mg/dL	Hepatitis A, B	Dengue	Platelets, $\times 10^3/\mu L$
1	Blood	Blood	Blood	412	1,483	0.7	2.7	Negative	NR	199
2	Urine	Urine	Urine	22	11	4.2	0.7	Negative	NR	220
3	Neg	Urine	Urine	1,160	1,260	0.6	0.5	Negative	Negative	228
4	Neg	Neg	Urine	280	670	1.9	7.6	Negative	NR	209

*PCR was used to determine the presence of *Leptospira* genes *lipL32* and *rrs*. NR, no results available: +, positive.

Appendix Table 10. Risk ratios for positive *Leptospira rrs* (16S rDNA) gene PCR according to symptoms in hospitalized patients*

Symptom/treatment	*rrs*-positive PCR, blood or urine	*rrs*-negative PCR, blood and urine	Risk ratio (95% CI)	p value†
Culture positive	35/37	1/34	17.04 (4.4–65.5)	**<0.001**
Conjunctival suffusion	25/37	10/33	2.08 (1.26–3.45)	**0.002**
Myalgias	28/37	14/34	2.15 (1.20–3.85)	**0.003**
Dyspnea	11/37	2/34	1.89 (1.31–2.73)	**0.009**
Cough	24/37	13/34	1.70 (1.04–2.76)	**0.025**
Hemoptysis	7/36	1/34	1.87 (1.29–2.72)	**0.030**
Antimicrobial drugs	21/31	11/24	1.51 (0.89–2.56)	0.102
Oliguria	9/35	4/33	1.46 (0.93–2.31)	0.15
Diarrhea	2/37	5/34	0.52 (0.16–1.72)	0.19
Vomiting	14/37	8/34	1.35 (0.88–2.09)	0.19
Death	8/36	0/6	1.23 (1.04–1.42)	0.20
Arthralgias	20/35	14/33	1.33 (0.83–2.14)	0.22
Dengue	3/21	5/17	0.63 (0.24–1.60)	0.25
Fever	36/37	31/34	2.15 (0.39–11.9)	0.26
Icterus	28/37	22/34	1.31 (0.75–2.27)	0.31
Rash	7/37	4/30	1.19 (0.71–1.98)	0.54
Hepatitis	4/23	4/16	0.82 (0.39–1.72)	0.56

*The *rrs*-positive and *rrs*-negative columns indicate the number of patients who had each feature over the total number of patients in each group for whom data were available.
†p values from Pearson's χ^2 test. Bold numbers indicate significant differences (p<0.05).

Appendix Table 11. Comparison of mean laboratory values from hospitalized patients who had positive versus negative PCR tests for the *Leptospira rrs* gene*

	rrs-positive, blood or urine	*rrs-negative,* blood and urine		No. patients,†
Laboratory test	Mean (95% CI)	Mean (95% CI)	p value	*rrs+/rrs–*
Potassium, mmol/L	3.8 (3.5–4.2)	4.1 (3.8–4.4)	0.17	34/34
Direct bilirubin, mg/dL	2.5 (1.6–3.3)	3.6 (2.0–5.2)	0.21	37/34
Creatinine, mg/dL	1.6 (0.7–2.4)	1.0 (0.8–1.2)	0.23	36/33
Indirect bilirubin, mg/dL	3.6 (1.3–3.4)	3.60 (1.6–5.6)	0.25	37/34
Hematocrit, %	35.7 (31.7–39.5)	38.2 (35.5–40.8)	0.29	36/34
AST, IU/L	394 (213–323)	503 (329–678)	0.38	37/34
Lymphocytes, %	22.7 (17.5–27.9)	20.2 (17.0–23.5)	0.41	27/28
Neutrophils, %	64.5 (57.5–71.4)	67.5 (60.4–74.5)	0.53	27/28
Urea, mg/dL	25 (17–33)	22 (13.3–30.4)	0.59	34/34
No. leukocytes, × $10^3/\mu L$	10.8 (8.6–13.0)	10.3 (8.3–12.2)	0.72	37/34
ALT, IU/L	585 (270–899)	561 (326–796)	0.90	37/34
No. platelets, × $10^3/\mu L$	243 (188–298)	243 192–293	0.99	37/34

*p values were calculated from 2-tailed *t*-tests. ALT, alanine aminotransferase; AST, aspartate aminotransferase; –, negative; +, positive.
†Number of patients (for whom data were available) who had a positive PCR test for the *Leptospira rrs* (16s rDNA) gene over the number of patients with a negative PCR test for *Leptospira rrs*.

Appendix Table 12. Risk ratios for fatal outcomes according to clinical features of hospitalized patients who had positive PCR tests for *Leptospira rrs* (16S rDNA) in either blood or urine*

Clinical feature	Survived, n = 29	Deceased, n = 8	Risk ratio (95% CI)	p value†
Oliguria	6/29	3/6	2.89 (0.71–11.83)	0.13
Hemoptysis	4/28	3/8	2.49 (0.77–8.00)	0.14
lipL32+ blood, PCR	10/29	5/8	2.44 (0.68–8.72)	0.15
Dyspnea	7/29	4/8	2.36 (0.72–7.79)	0.16
Icterus	21/29	7/8	2.25 (0.32–15.9)	0.38
rrs+ blood, PCR	12/29	5/8	1.96 (0.54–7.03)	0.29
Antimicrobial drug treatment	17/26	4/5	1.90 (0.24–14.9)	0.52
lipL32+ urine, PCR	10/29	3/8	1.11 (0.31–3.91)	0.87
Cough	19/29	5/8	0.90 (0.25–3.19)	0.87
Conjunctival suffusion	20/29	5/8	0.80 (0.23–2.81)	0.73
Blood culture positive	13/29	3/8	0.79 (0.22–2.82)	0.71
Arthralgias	17/29	3/6	0.75 (0.17–3.21)	0.70
Urine culture positive	18/29	4/8	0.68 (0.20–2.31)	0.54
rrs+ urine, PCR	18/29	4/8	0.68 (0.20–2.31)	0.54
Rash	6/29	1/8	0.61 (0.89–4.21)	0.60
Vomiting	12/29	2/8	0.55 (0.13–2.35)	0.40
Culture positive	28/29	7/8	0.40 (0.09–1.86)	0.32
Myalgias	24/29	4/8	0.32 (0.10–1.03)	**0.06**
Fever	29/29	7/8	0.19 (0.10–0.38)	**0.05**
Abdominal pain	21/29	1/8	0.10 (0.13 - 0.71)	**0.002**
Diarrhea	2/29	0/8	0	0.44

*Survived and deceased columns show the number of patients with each feature over the total number of patients in each group for whom data were available. PCR detected *Leptospira lipL32* and *rrs* genes. +, positive.

†p values were calculated from Pearson's χ^2 tests. Bolded p values indicate significant differences ($p<0.05$).

Appendix Table 13. Comparison of mean laboratory values in surviving versus deceased hospitalized patients who had positive PCR tests for *Leptospira rrs* (16S rDNA) in either blood or urine*

	Survived, n = 29	Deceased, n = 8			
Laboratory test	Mean (95% CI)	Mean (95% CI)	p value	No. deceased/ no. survived†	Cohen's d (95% CI)
Creatinine, mg/dL	1.1 (0.8–1.4)	3.1 (–1 to 7.4)	0.06	8/28	–0.77 (–1.57 to 0.04)
Urea, mg/dL	18.6 (13.4–23.8)	45.6 (16.4–74.8)	0.07	8/26	–1.36 (–2.21 to –0.49
No. leukocytes, × $10^3/\mu L$	9.9 (7.6–12)	14.3 (7.2–21.3)	0.10	8/29	–0.68 (–1.47 to 0.12)
ALT, IU/L	705 (311–1,097)	152 (5–299)	0.14	8/29	0.60 (–0.20 to 1.39)
Neutrophils, %	61.8 (55–69)	73.5 (49–98)	0.15	6/21	–0.68 (–1.60 to 0.25)
Lymphocytes, %	24.3 (19.3–29)	17.1 (–3 to 37)	0.25	6/21	–0.55 (–0.38 to 1.46)
AST, IU/L	448 (221–674)	202 (15–389)	0.26	8/29	0.45 (–0.34 to 1.24)
Potassium, mmol/L	3.7 (3.4–4.1)	4.1 (2.9–5.3)	0.33	8/26	–0.40 (–1.20 to 0.40)
No. platelets, × $10^3/\mu L$	257 (190–325)	193 (106–280)	0.33	8/29	0.39 (–0.40 to 1.18)
Hematocrit, %	36.4 (31.8–41.0)	32.7 (25–41)	0.45	7/29	0.32 (–0.51 to 1.15)
Direct bilirubin, mg/dL	2.6 (1.5–3.6)	2.17 (0.3–4.0)	0.72	8/29	0.14 (–0.64 to 0.92)
Indirect bilirubin, mg/dL	2.3 (1.1–3.5)	2.5 (0.1–4.9)	0.87	8/29	–0.07 (–0.85 to 0.72)

*p values were calculated from 2-tailed *t*-tests. ALT, alanine aminotransferase; AST, aspartate aminotransferase.

†No. deceased/no. survived indicates the number of patients in each group for whom data were available.

Appendix Table 14. Microscopic agglutination test antibody titers in serum samples from dairy cows at 1 farm*

	Serovars‡																						
Cow no., culture†	a	b	c	d	e	f	g	h	i	j	k	l	m	n	ñ	o	p	q	r	s	t	u	v
1, *L. venezuelensis*	–	–	–	–	–	–	–	–	–	–	–	–	–	–	–	–	–	–	–	–	–	–	–
2	–	–	–	–	–	–	–	–	–	–	–	–	–	–	–	–	–	–	–	–	–	–	–
3, *L. venezuelensis*	–	–	1:100	–	–	1:100	–	–	–	–	–	–	–	–	–	–	1:800	–	1:100	–	–	1:200	–
4	–	–	–	–	–	–	–	–	–	–	–	–	–	–	–	–	–	–	–	–	–	–	–
5, *L. interrogans*	–	–	–	–	–	–	–	–	–	1:400	–	–	–	1:200	-	–	–	–	1:400	–	–	–	–
6	–	–	1:50	–	–	–	–	–	–	–	–	–	–	–	–	–	–	–	–	–	–	–	–
7, *L. venezuelensis*	–	–	–	–	–	1:200	1:400	–	–	–	–	–	–	–	–	–	1:800	-	1:400	–	–	1:100	–
8, *L. interrogans*	–	–	1:50	–	–	–	–	–	–	–	–	–	–	–	–	–	–	–	–	–	–	–	–
9, *L. interrogans*	–	–	–	–	–	1:200	–	–	–	–	1:200	–	–	–	1:50	-	1:100	1:100	1:200	–	–	1:400	–
10	–	–	–	–	–	–	–	–	–	–	-	–	–	–	–	–	–	–	–	–	–	–	–
11	–	–	1:50	1:1,600	–	–	–	–	–	–	1:100	–	–	–	–	–	1:200	1:50	1:200	–	–	1:400	–
12, *L. venezuelensis*	–	–	–	–	–	1:100	–	–	–	–	1:100	–	–	–	1:100	-	1:800	–	–	–	–	–	–
13, *L. venezuelensis*	–	–	1:100	–	–	1:100	–	–	–	–	1:100	–	–	–	–	–	1:400	-	1:200	–	–	–	–
14, *L. venezuelensis*	1:50	–	1:50	–	–	1:200	1:200	–	–	–	1:50	–	–	–	–	–	1:800	1:50	1:200	–	–	1:400	–
15	1:50	–	–	–	–	–	–	–	–	–	–	–	–	–	–	–	–	–	–	–	–	–	–
16, *L. venezuelensis*	–	–	1:50	–	–	–	–	–	–	1:100	–	1:200	–	–	–	–	1:400	–	1:400	–	–	–	–

*Antibody titers were determined by using live antigens of 23 reference *Leptospira* serovars. –, negative.
†*Leptospira* species isolated from urine cultures. Sixteen cows were tested, only 10 had *Leptospira*-positive cultures.
‡*Leptospira* serovars were: a, *L. javanica*; b, *L. grippothyphosa*; c, *L. hurstibridge*; d, *L. bataviae*; e, *L. autumnalis*; f, *L. hardjo*; g, *L. tarassovi*; h, *L. pomona*; i, *L.* celledoni; j, *L. canicola*; k, *L. sejroe;* l, *L. pyrogenes*; m, *L. icterohaemorrhagiae* RGA; n, *L. icterohaemorrhagiae* 3294; ñ, *L. copenhageni*; o, *L. shermani*; p, *L. wolffi*; q, *L. castelonis*; r, *L. hebdomadis*; s, *L. muenchen*; t, *L. cynoptery*; u, *L. mini*; v, *L. panama*.

Appendix Table 15. Comparison of VNTR loci and MLST alleles in *L. interrogans* strains isolated from hospitalized patients or rodents in La Guaira, Venezuela

	VNTR loci†				MLST alleles†						
Isolates*	VNTR4	VNTR7	VNTR10	VNTR-Lb5	*glmU*	*pntA*	*sucA*	*tpiA*	*pfkB*	*mreA*	*caiB*
CAB-H41	3	9	16	6	1	1	2	1	7	7	8
CAY-U48	1	9	3	NR	1	1	2	1	7	4	3
CAB-U03	3	NR	2	5	1	1	2	2	7	4	3
MAC-H04	2	NR	15	NR	1	1	2	2	7	4	5
CLM-R09-A	1	1	3	NR	1	1	2	2	7	4	8
CLM-H09	1	1	3	NR	1	1	3	2	4	7	5
URI-U06	3	2	NR	3	1	1	3	2	7	4	3
URI-H01	3	9	13	4	1	1	3	2	7	4	3
CLM-R11-A	2	1	7	5	1	1	3	3	4	6	19
CLM-U30	2	9	2	6	1	3	2	2	4	4	19
MAC-H63	2	9	NR	NR	1	3	2	2	7	7	19
CAY-H65	2	0	16	5	1	3	3	1	4	5	5
SOB-U13	3	9	14	NR	1	12	3	3	10	4	5
CLM-U22	3	12	7	NR	1	12	2	3	10	6	19
MAQ-U18	2	1	7	5	1	12	3	3	10	5	19
CLM-U28	1	11	3	4	1	12	3	3	10	6	19
GUA-H40	2	8	9	4	1	12	3	3	10	6	19
CLM-H08	3	12	9	NR	1	12	3	3	10	6	19
SOB-R13-B	3	9	12	NR	1	12	3	3	10	6	19
CLM-U45	2	10	3	NR	3	3	3	2	4	5	5
CLM-U47	2	15	NR	NR	3	3	3	3	4	5	5
NAG-U02	3	1	12	16	6	1	3	2	4	7	3
CAY-U49	2	4	16	6	6	1	3	3	76	7	3
CLM-U24	1	9	4	6	6	1	3	12	4	5	5
CLM-U46	2	NR	NR	4	6	2	3	3	7	7	19
GUA-H52	2	5	6	4	6	3	2	2	4	4	3
GUA-H64	1	9	15	5	6	3	2	3	4	7	5
CAB-U11	2	1	7	5	6	3	3	2	4	5	5
GUA-H21	2	9	3	5	6	3	3	3	1	7	5
CAO-U23	2	1	NR	NR	6	3	3	3	4	5	19
MAQ-H53	1	9	6	4	6	8	2	2	9	7	5
MAQ-H60	2	NR	2	8	6	8	2	2	9	7	5
GUA-R52	1	9	10	3	6	NA	NA	NA	NA	4	NA
URI-R01-B	0	3	NR	NR	NA	NA	NA	NA	NA	NA	NA
URI-R01-A	1	3	NR	NR	NA	NA	NA	NA	NA	NA	NA
GUA-R21	1	8	11	3	NA	NA	NA	NA	NA	NA	NA
CLM-R48-B	2	NR	1	NR	NA	NA	NA	NA	NA	NA	NA
CLM-R48-A	2	NR	3	7	NA	NA	NA	NA	NA	NA	NA
URI-R06-B	2	1	10	NR	NA	NA	NA	NA	NA	NA	NA
CLM-R46	2	1	10	2	NA	NA	NA	NA	NA	NA	NA
CAB-R03	2	10	NR	NR	NA	NA	NA	NA	NA	NA	NA
CLM-R11-B	2	10	17	6	NA	NA	NA	NA	NA	NA	NA
CLM-R22	2	12	10	3	NA	NA	NA	NA	NA	NA	NA
MAQ-R53	2	2	11	4	NA	NA	NA	NA	NA	NA	NA
CLM-R09-B	2	3	3	NR	NA	NA	NA	NA	NA	NA	NA
CLM-R09-C	2	3	3	NR	NA	NA	NA	NA	NA	NA	NA
URI-R01-D	2	9	16	6	NA	NA	NA	NA	NA	NA	NA
CLM-R08	2	9	3	5	NA	NA	NA	NA	NA	NA	NA
CAY-R49	3	NR	11	NR	NA	NA	NA	NA	NA	NA	NA
SOB-R13-C	3	0	NR	NR	NA	NA	NA	NA	NA	NA	NA
URI-R06-A	3	1	NR	4	NA	NA	NA	NA	NA	NA	NA
SOB-R13-A	3	2	12	5	NA	NA	NA	NA	NA	NA	NA
CLM-R45	3	3	NR	7	NA	NA	NA	NA	NA	NA	NA
CAY-R65	3	4	NR	NR	NA	NA	NA	NA	NA	NA	NA
MAC-R63	4	5	8	NR	NA	NA	NA	NA	NA	NA	NA
GUA-R64	8	3	10	NR	NA	NA	NA	NA	NA	NA	NA
URI-R01-C	8	3	3	5	NA	NA	NA	NA	NA	NA	NA

*The first 3 letters describing each isolate indicate the area of the patient's residence or where the rodent was captured in the state of La Guaira: CAB, Caraballeda; CAO, Caruao; CAY, Carayaca; CLM, Catia La Mar; GUA, La Guaira; MAC, Macuto; MAQ, Maiquetia; NAG, Niguata; SOB, Soublette; or URI, Urimare. The last 3 letters begin with H (isolated from human blood), R (isolated from rat tissue), or U (isolated from human urine). Rodent isolates are underlined. MLST, multilocus sequence typing; NA, not applicable; NR, no results for this locus;.VNTR, variable number of tandem repeats.

†Shading indicates clustered profiles that are identical, differ from each other by 1 allele. MLST profiles were only obtained for those rodent isolates with VNTR patterns similar to ≥1 patient isolate.

Appendix Table 16. VNTR typing of 3 *L. interrogans* strains isolated from dairy cows at 1 farm*

	No. copies of 4 VNTR loci			
Cow no.	VNTR4	VNTR7	VNTR10	VNTR-Lb5
5	2	9	13	3
8	2	9	13	3
9	3	11	9	4

*Shading indicates identical VNTR profiles. VNTR, variable number of tandem repeat.
